# A review of cardiorespiratory fitness-related neuroplasticity in the aging brain

**DOI:** 10.3389/fnagi.2013.00031

**Published:** 2013-07-12

**Authors:** Scott M. Hayes, Jasmeet P. Hayes, Margaret Cadden, Mieke Verfaellie

**Affiliations:** ^1^Memory Disorders Research Center, VA Boston Healthcare System, Boston University School of MedicineBoston, MA, USA; ^2^Neuroimaging Research for Veterans Center, VA Boston Healthcare SystemBoston, MA, USA; ^3^Department of Psychiatry, Boston University School of MedicineBoston, MA, USA; ^4^National Center for Posttraumatic Stress Disorder, VA Boston Healthcare SystemBoston, MA, USA

**Keywords:** exercise, physical fitness, physical activity, fMRI, structural MRI, diffusion tensor imaging, episodic memory, executive functions

## Abstract

The literature examining the relationship between cardiorespiratory fitness and the brain in older adults has increased rapidly, with 30 of 34 studies published since 2008. Here we review cross-sectional and exercise intervention studies in older adults examining the relationship between cardiorespiratory fitness and brain structure and function, typically assessed using Magnetic Resonance Imaging (MRI). Studies of patients with Alzheimer's disease are discussed when available. The structural MRI studies revealed a consistent positive relationship between cardiorespiratory fitness and brain volume in cortical regions including anterior cingulate, lateral prefrontal, and lateral parietal cortex. Support for a positive relationship between cardiorespiratory fitness and medial temporal lobe volume was less consistent, although evident when a region-of-interest approach was implemented. In fMRI studies, cardiorespiratory fitness in older adults was associated with activation in similar regions as those identified in the structural studies, including anterior cingulate, lateral prefrontal, and lateral parietal cortex, despite heterogeneity among the functional tasks implemented. This comprehensive review highlights the overlap in brain regions showing a positive relationship with cardiorespiratory fitness in both structural and functional imaging modalities. The findings suggest that aerobic exercise and cardiorespiratory fitness contribute to healthy brain aging, although additional studies in Alzheimer's disease are needed.

## Introduction

Approximately 5.3 million Americans currently suffer from Alzheimer's disease (AD; Hebert et al., 2003; Mebane-Sims and Association, 2009), and some models forecast that the prevalence of AD will quadruple within the U.S. and worldwide within the next 50 years (Brookmeyer et al., 1998, 2007). The alarming increase in AD prevalence presents an ever-increasing burden on families, caregivers, and healthcare systems. A cure for AD has yet to be identified, underscoring the need for interventions that prolong cognitive function and independence prior to disease onset, thereby reducing caregiver burden, stress, and healthcare costs.

Aerobic exercise programs represent a low-cost, large-scale behavioral intervention that may slow the progression of cognitive and neural decline in healthy older adults. Certainly aerobic exercise does not represent a cure for AD, but it has been suggested that such programs may have the potential to delay the onset of AD (Rovio et al., 2005; Foster et al., 2011). Animal studies of aging and AD have demonstrated that physical activity has a positive impact on the brain, including attenuation of age-related decreases in hippocampal neurogenesis (Van Praag et al., 2005; Kronenberg et al., 2006), reductions in levels of amyloid-beta (Aβ), a neuropathological marker of AD (Adlard et al., 2005) and pro-inflammatory cytokines (for review, see Cotman et al., 2007), and enhanced levels of brain-derived neurotrophic factor (which supports neurogenesis) and synaptophysin (a marker of synaptic function) in rodents at genetic risk for AD (Nichol et al., 2009); for review, see (Cotman et al., 2007; Van Praag, 2009; Lista and Sorrentino, 2010). Meta-analytic studies in humans have provided evidence for a positive relationship between aerobic exercise training and cognitive function in healthy older adults (Colcombe and Kramer, 2003; but see Etnier et al., 2006) and benefits in memory subsequent to exercise intervention in patients with mild cognitive impairment (MCI), who are at risk for development of AD (Smith et al., 2010) and dementia (Heyn et al., 2004, 2008). Moreover, meta-analytic results have indicated that the cognitive benefits of exercise in older adults with MCI are larger than those observed in cognitively intact adults (Smith et al., 2010). These results, in combination with the finding that moderate levels of exercise in mid- and late-life are associated with decreased risk for MCI (Geda et al., 2010), suggest that physical activity may attenuate age-related decline and influence the development of AD (Fratiglioni et al., 2004; Rovio et al., 2005).

Recent structural and functional Magnetic Resonance Imaging (fMRI) studies have provided support for a positive relationship between cardiorespiratory fitness and brain structure and function in humans. An examination of this nascent literature, which has seen a tripling in the number of peer-reviewed studies since 2008, may provide insight into brain regions most likely to be influenced by exercise in humans. Recent brief reviews on this topic have targeted children and older adults (Voss et al., 2011) or genetic and dietary moderators of the relationship between physical activity and the brain (Leckie et al., 2012). Here we present a comprehensive review of structural and functional neuroimaging studies (MRI or PET) focusing exclusively on healthy older adults and, when available, discuss related studies of AD. We completed a literature search on PubMed and Web of Knowledge using search terms including “fitness,” “physical activity,” or “exercise” and “MRI” or “PET.” Criteria for inclusion in the review were as follows: 1) human subjects with mean age of 55 years or older 2) neuroimaging with MRI or PET 3) measurement of cardiorespiratory fitness either alone (e.g., VO_2_ max) or as part of a composite measure of physical fitness (e.g., using VO_2_ max and hand grip strength), or an estimate of cardiorespiratory fitness, either derived from equations using age, gender, body mass index or inferred from self-report physical activity questionnaires (that focused on aerobic activities such as walking). Reference sections of relevant articles were also examined to identify additional studies.

At the outset of this review, we provide a brief discussion of the concepts of physical activity, exercise, and fitness and their relationship. This section is followed by a comprehensive review of evidence in humans that cardiorespiratory fitness is positively associated with (1) brain structure and (2) brain function. Within each of these sections, we first review cross-sectional studies, followed by exercise intervention studies. Given that the medial temporal lobes (MTL), specifically the hippocampus, is one of the primary targets of physical activity effects in the rodent brain (for review, Cotman et al., 2007; Van Praag, 2008) and a primary target of age- and AD-related neurodegeneration in humans (Braak and Braak, 1991; Jack et al., 2002; Raz et al., 2005), we highlight MTL results when available. We conclude with a discussion focusing on challenges facing the field and directions for future research.

## Physical activity, exercise, and fitness

**Figure 1:**
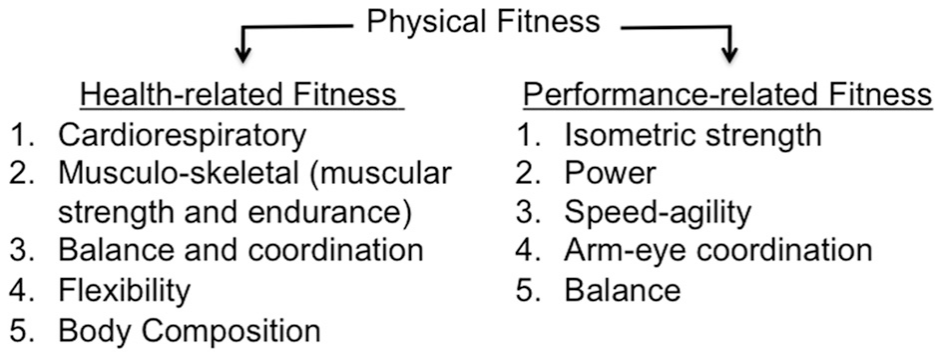
**Taxonomy of physical fitness**.

The terms physical activity, exercise, and fitness are often used interchangeably but represent distinct constructs. Physical activity has been defined as “any bodily movement produced by skeletal muscle that results in energy expenditure” (Caspersen et al., 1985, p. 126). Physical activities range in the amount of energy expenditure and encompass activities as varied as leisure (e.g., watching TV, playing basketball), school/occupational (e.g., walking a study participant to the MRI scanner, unloading boxes from a delivery truck), household/domestic/self-care (e.g., rocking a baby, mowing the lawn), or transportation (e.g., sitting in the car, walking to work). Exercise shares characteristics of physical activity (bodily movement by skeletal muscles that expends energy), but it is considered a distinct sub-category that it is “planned, structured, repetitive and purposive in the sense that improvement or maintenance of one or more components of physical fitness is an objective” (Caspersen et al., 1985, p. 128). For example, playing basketball once every 3 weeks for 2 h would be considered physical activity (but not exercise), whereas playing 1 h three times per week for 8 weeks with the goal of improving fitness would be considered exercise. Physical activity and exercise refer to bodily movement; by contrast, physical fitness is “a set of attributes that people have or achieve” (Caspersen et al., 1985, p. 128) and is defined as “the ability to carry out daily tasks with vigor and alertness, without undue fatigue and with ample energy to enjoy leisure time pursuits and to meet unforeseen emergencies.” Fitness is categorized as performance-related (abilities associated with athletic performance) or health-related (traits associated with reduced risk of hypokinetic diseases such as obesity, type-II diabetes, cardiovascular disease, heart disease; see Figure 1).

Interest in the relationship between physical fitness and brain health has centered on health-related fitness, and specifically cardiorespiratory fitness. Cardiorespiratory fitness refers to the ability to perform large-muscle, dynamic, moderate to high intensity, physical activity for a prolonged period, and depends on the integrity of the cardiovascular, respiratory, and skeletal muscle systems. The focus on cardiorespiratory fitness in neuroimaging studies of humans is attributable to work linking cardiorespiratory fitness-enhancing exercise (wheel-running) in rodents with upregulation of neurotrophins (e.g., brain-derived neurotrophic factor, BDNF), neurovascular plasticity (primarily via angiogenesis), and neurogenesis, as well as human studies linking aerobic training to enhanced cognition (e.g., Churchill et al., 2002). Cardiorespiratory fitness has been assessed in a variety of ways. Measures of oxygen utilization during a graded treadmill test provide a direct index of cardiorespiratory fitness, and are considered the gold standard. In such tests, treadmill speed and grade increase at 1–2 min intervals and the participant's volume of oxygen (VO_2_) consumption is measured throughout the test. Relevant measures include VO_2_ peak (highest volume of oxygen value attained on a graded exercise test) and VO_2_ max (value at which VO_2_ plateaus or increases minimally despite increased workload on graded exercise test). Cycle ergometer protocols are also used, although peak VO_2_ values tend to be 10–20% below those obtained using a treadmill protocol (Miyamura and Honda, 1972). Lacking a maximal VO_2_ treadmill or cycle ergometer test, some neuroimaging studies have implemented sub-maximal estimates of VO_2_ using the Rockport one-mile walk test (correlations as high as 0.88 with graded treadmill measures of VO_2_, e.g., Colcombe et al., 2004) or have estimated cardiorespiratory fitness based on equations using variables such as age and body mass index, information that is more easily available.

In the absence of direct measures of cardiorespiratory fitness, physical activity questionnaires that probe one's physical activities over a period of time (e.g., how far did you walk 6 days ago?) have been used in a number of neuroimaging studies. Use of such questionnaires is based on the assumption that subjects who report greater amounts of physical activity are also likely to have higher physical fitness (although it is important to keep in mind that factors such as genetics and nutrition also impact fitness). The questionnaire approach is subject to reporting inaccuracies, omissions and bias, and misunderstanding questions (Rzewnicki et al., 2003; Shepard, 2003), issues that may be exacerbated in older adults with age-related memory decline and those at risk for AD. Recently developed technologies such as accelerometers, which estimate energy expenditure based on measurement of movements and vector magnitudes in three directions (Freedson et al., 1998, 2012), provide an objective measure of physical activity independent of self-report, but this approach has yet to be implemented in neuroimaging studies with older adults. Nevertheless, there is evidence of moderate correlations between the results of subjective self-report physical activity questionnaires and objective VO_2_ max values based on a graded treadmill test (Bowles et al., 2004). Additionally, similar effects of cardiorespiratory fitness on cognitive and neural measures have been obtained in older adults, regardless of the method of fitness assessment (McAuley et al., 2011; but see Floel et al., 2010). Therefore, in our current review, we include studies that used self-report physical activity questionnaires, with the caveat that this approach relies on the inference that those with higher activity levels are likely to have higher fitness levels. Importantly, the questionnaires implemented in the reported studies focused specifically on physical activities such as walking, running and jogging (Taylor et al., 1978; Bowles et al., 2004; Taylor-Piliae et al., 2006) that link to cardiorespiratory fitness, or researchers extracted items from broader assessments of physical activity (Stewart et al., 2001) to primarily focus on those activities that would impact cardiorespiratory fitness (e.g., walking, biking, water exercises).

Our review revealed a limited number of studies implementing the same fitness assessment techniques. A primary goal is to give a comprehensive review of neuroimaging studies examining cardiorespiratory fitness (either directly or inferred via physical activity questionnaire) in older adults. We considered studies using any of the fitness assessment techniques previously described and studies implementing aerobic exercise training aimed at enhancing cardiorespiratory fitness (either alone or in combination with another training modality, e.g., muscular strength). We note the specific fitness assessment methods for each study in Tables 1, 2. When the primary measure of cardiorespiratory fitness (VO_2_ max or VO_2_ peak) is assessed with methods other than the gold standard (maximal test using treadmill or cycle ergometer), the method of assessment is noted in the text. Finally, we note that there is a difference in the strength of the conclusions to be made for cross-sectional relative to intervention studies, with the primary caveat that cross-sectional relationships do not imply causation. That is, the cross-sectional studies indicate whether neural differences exist within subgroups of older adults (high vs. low cardiorespiratory fitness). Although observed brain differences are associated with cardiorespiratory fitness, it is possible that a number of unstudied factors may also influence the results, such as genetics, diet, blood pressure, etc. Indeed, results from cross-sectional studies should be interpreted with caution for this reason. Despite these potential issues, results from the cross-sectional studies were relatively consistent with findings from the intervention studies and therefore will be discussed in this review.

**Table 1:** **Cross-sectional (CS) and exercise intervention (INVN) studies examining the relationship between cardiorespiratory fitness and brain structure**.

**Author**	**Year**	**Study type**	**Imaging modality**	**Analysis**	**Fitness measure**	**Group**	**Subjects (Number of females)**	**Mean age (years)**
Bugg and Head	2011	CS	T1	ROI	Questionnaire	OA	52 (37)	69
Burns et al.	2008	CS	T1	Whole brain	VO_2_ peak Questionnaire	Early AD	57 (31)	74
						OA	64 (34)	73
Colcombe et al.	2003	CS	T1	Whole brain	Rockport 1 mile walk	OA	55 (31)	66
Erickson et al.	2009	CS	T1	ROI	VO_2_ peak	OA	165 (109)	67
Erickson et al.	2010	CS	T1	Whole brain	Total number of blocks walked during 1 week	OA	299 (182)	78
Floel et al.	2010	CS	T1	Whole brain	Ergometer test, Lactate step test, Questionnaire	OA	75 (47)	60
Gordon et al.	2008	CS	T1	Whole brain	VO_2_ max	YA	20 (10)	22
						OA	40 (23)	72
Ho et al.	2011	CS	T1	Whole brain ROI	Questionnaire	OA	226 (130)	78
Honea et al.	2009	CS	T1	Whole brain	VO_2_ peak, Questionnaire	Early AD	61 (37)	74
						OA	56 (33)	73
McAuley et al.	2011	CS	T1	ROI	VO_2_ peak, Rockport 1 mile walk	OA	86 (53)	65
Sen et al.	2012	CS	T1	Whole brain	Bicycle ergometer test	OA	715 (386)	65
Szabo et al.	2011	CS	T1	ROI	VO_2_ peak, Questionnaire	OA	158 (105)	66
Verstynen et al.	2012	CS	T1	ROI	VO_2_ max	OA	179 (109)	67
Vidoni et al.	2012	CS	T1	ROI	VO_2_ peak	AD	37 (20)^*^	74
						OA	53 (29)	73
Weinstein et al.	2012	CS	T1	ROI	VO_2_ max	OA	142 (91)^+^	67
Marks et al.	2011	CS	DTI	ROI	VO_2_ peak, self report of activity per week	OA	15 (7)	66
Johnson et al.	2012	CS	DTI	Whole brain	Composite score: VO_2_ peak, total time on treadmill, 1 minute heart rate recovery	OA	26 (14)	65
Marks et al.	2007	CS	DTI	ROI	Equation derived estimate	YA	13	24
						OA	15	70
Head et al.	2012	CS	PiB	ROI	Questionnaire	OA	163	45–88
Liang et al.	2010	CS	PiB	ROI	Questionnaire	OA	54	55–88
Colcombe et al.	2006	INVN	T1	Whole brain	VO_2_ peak	OA	59	66
Erickson et al.	2011	INVN	T1	ROI	VO_2_ max	OA	120	55–80
Ruscheweyh et al.	2011	INVN	T1	Whole brain	Ergometer test, Questionnaire, Lactate step test	OA	62 (43)	60
Voss et al.	2012	INVN	DTI	ROI	Composite score: VO_2_ max, Rockport 1 mile walk	OA	70 (45)	65

AD, Alzheimer's disease; DTI, diffusion tensor imaging; OA, older adults; PiB, Pittsburgh Compound B amyloid imaging; ROI, regions of interest analysis; T1, T1-weighted imaging; YA, young adults.

* Nine subjects from each group excluded from T1 analysis, gender distribution of excluded subjects unknown.

+ 139 participants completed the spatial working memory task.

**Table 2:** **Cross-sectional (CS) and exercise intervention (INVN) studies examining the relationship between cardiorespiratory fitness and brain function (all studies used fMRI)**.

**Author**	**Year**	**Study type**	**Cognitive domain**	**Functional task**	**Fitness measure**	**Group**	**Number of subjects (females)**	**Mean age (years)**
Colcombe et al.	2004	CS	Executive functions	Flanker task	Rockport 1 mile walk	OA	41	Fit: 66
								Sed: 68
Godde and Voelcker-Rehage	2010	CS	Motor	Imaginary motor task	Composite score: hand tapping, feet tapping, balance, maximal grip strength and mean VO_2_	OA	51 (38)	69
Prakash et al.	2011	CS	Executive functions	Stroop task	Composite score: VO_2_ max and Rockport 1 mile walk	OA	70	65
Rosano et al.^*^	2010	CS	Executive functions	Digit symbol substitution task	Questionnaire	OA	Fit: 17	81
							Sed: 10	81
Smith et al.	2011a	CS	Semantic memory	Famous name recognition task	Questionnaire	OA	68 (50)	73
Smith et al.	2011b	CS	Semantic memory	Famous name recognition task	Questionnaire	aMCI	18 (14)	Fit: 75
								Sed: 74
McGregor et al.	2011	CS	Motor	Finger tapping	Aerobic endurance test, Questionnaire	YA	YA: 12 (5)	24
						OA	Fit OA: 12 (6)	68
							Sed OA: 12 (7)	71
Voss et al.	2010a	CS	N/A	Passive viewing task	VO_2_ max	YA	32 (27)	24
						OA	120 (85)	66
Colcombe et al.	2004	INVN	Executive functions	Flanker task	VO_2_ peak	OA	29	58–77
Voelcker-Rehage et al.	2011	INVN	Executive functions	Flanker task	Composite score: VO_2_ peak, action speed, reaction speed, and balance	OA	44 (28)	70
Voss et al.	2010b	INVN	N/A	Passive viewing task	VO_2_ max	YA	YA: 32	24
						OA	OA: 35 (Control)	65
							OA: 30 (INVN)	67

aMCI, amnestic mild cognitive impairment; N/A, not applicable; OA, older adults; Sed, Sedentary; YA, young adults.

* Exercise intervention, but MRI was completed 2 years post-training.

## Linking cardiorespiratory fitness to brain structure

To measure brain structure, voxel-based morphometry (VBM) is the most common analysis approach implemented in neuroimaging studies of fitness and brain structure (see Table 1). This automated approach segments the brain of each individual into gray matter, white matter, and cerebrospinal fluid maps, followed by normalization to a standard brain template for volumetric and group analysis of the various tissue types (Ashburner and Friston, 2000).

### Cross-sectional studies

**Figure 2:**
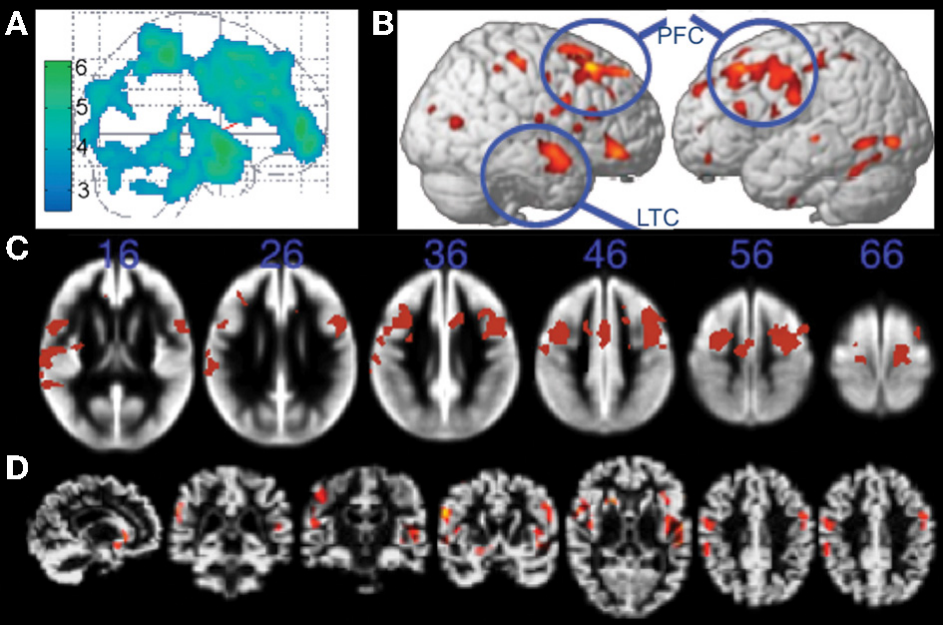
**Structural MRI studies showing positive relationships with brain volume and fitness. (A)** Gray matter regions, including prefrontal cortex and parietal regions, showing fitness-related preservation in older adults. From Colcombe et al. (2003), Figure 1, p. 178. Adapted with permission. **(B)** Regions showing increased brain volume in older adults who walked more than 72 blocks per week. From Erickson et al. (2010), Figure 2B, p. 1419. Adapted with permission. **(C)** Gray matter regions, including bilateral prefrontal cortex, showing a positive relationship with fitness (after controlling for age, gender, and education) in older adults. The blue numbers represent MNI coordinates in the axial (z) plane. From Weinstein et al. (2012), Figure 1A, p. 815. Adapted with permission. **(D)** Brain regions showing a positive relationship with fitness (after controlling for age, education, and gender) in older adults. From Gordon et al. (2008), Figure 3A, p. 835. Adapted with permission. LTC, lateral temporal cortex; PFC, prefrontal cortex.

In a seminal study, Colcombe and colleagues demonstrated using VBM that cardiorespiratory fitness (estimated VO_2_ from Rockport one-mile protocol) was associated with attenuated age-related decline in tissue density in fronto-parietal white and gray matter regions including prefrontal cortex, superior parietal cortex, and temporal cortex in healthy older adults (Figure 2A; Colcombe et al., 2003). Subsequent studies using treadmill based VO_2_ max have also reported positive associations between fitness and gray matter volume in ventrolateral and dorsolateral prefrontal cortex (Gordon et al., 2008; Weinstein et al., 2012), lateral parietal regions (Gordon et al., 2008; Weinstein et al., 2012), and MTL regions (Gordon et al., 2008). Furthermore, weekly walking distance (assessed via self-report questionnaire), was associated 9 years later with reduced risk of cognitive impairment and increased brain volume in lateral and medial prefrontal cortex, lateral temporal cortex, lateral parietal regions, and the MTL (Erickson et al., 2010). Greater brain volume in these same regions (Figure 2B) was associated with the highest quartile of walking distance, suggesting one should walk at least 72 blocks or more per week for beneficial brain effects (although additional benefit was not observed for those walking substantially more than 72 blocks; Erickson et al., 2010).

The exact overlap of brain regions across these studies is somewhat difficult to ascertain because two of the studies do not report coordinates in standard space (Colcombe et al., 2003; Gordon et al., 2008), and the effects are visually represented on different brain templates across the various studies (glass brain, gray matter segmentation, cortical surface rendering; Figures 2A–D). Nevertheless, visual inspection of the figures suggests that the cortical gray matter regions associated with cardiorespiratory fitness in cognitively intact older adults are relatively consistent, and include prefrontal, anterior cingulate, lateral parietal, and lateral temporal cortex (but see null results reported by Honea et al., 2009; Sen et al., 2012).

**Figure 3:**
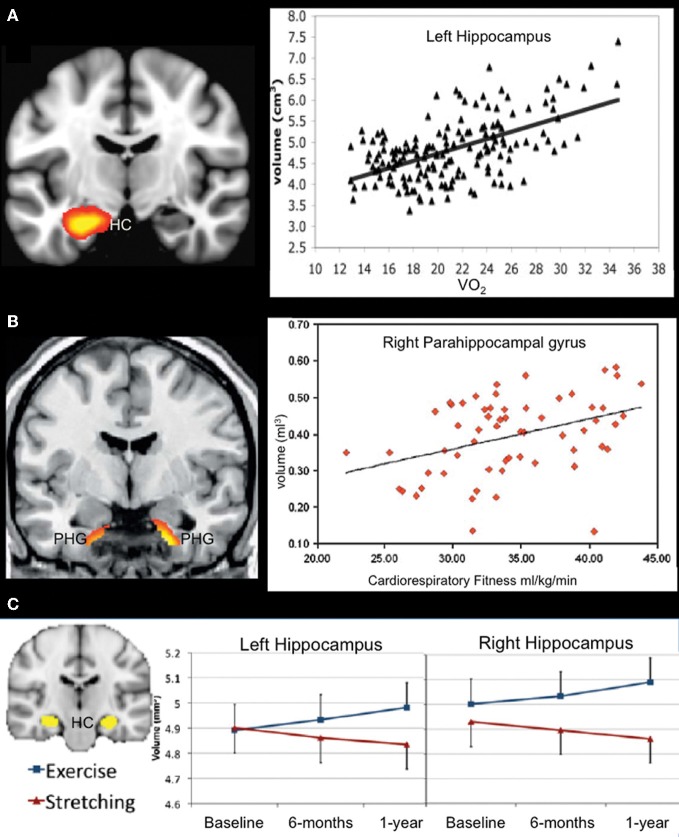
**Structural MRI studies using a regions-of-interest approach showing a positive relationship between fitness levels and medial temporal lobe volume. (A)** Aerobic fitness is associated with bilateral hippocampal volume in older adults (only data from left hippocampus are displayed). From Erickson et al. (2009), Figure 2, p. 1034. Adapted with permission. **(B)** Increased parahippocampal volume is associated with aerobic fitness in early AD patients. From Honea et al. (2009), Figure 3B, p. 194. Adapted with permission. **(C)** Aerobic exercise training increases bilateral hippocampal volume in older adults. From Erickson et al. (2011), Figure 1A, p. 3019. Adapted with permission. HC, hippocampus; PHG, parahippocampal gyrus.

Evidence for a relationship between cardiorespiratory fitness and the MTL (hippocampus, parahippocampal gyrus, and amygdala) in whole-brain VBM studies of older adults has been inconsistent, with only Gordon et al. (2008) and Erickson et al. (2010) reporting significant effects. However, region-of-interest (ROI) analyses focusing on MTL structures have yielded additional positive results. For instance, VO_2_ peak was associated with both left and right hippocampal volume in older adults (Figure 3A; Erickson et al., 2009). In another study, engagement in cardiorespiratory fitness-enhancing physical activities (assessed with a self-report questionnaire) was associated with reduced age-related volume decline in the MTL (Bugg and Head, 2011). This effect was primarily driven by the amygdala and parahippocampal gyrus rather than the hippocampus, although the effect was in the expected direction for the hippocampus. The inconsistent MTL findings across VBM and ROI studies could be attributed to differences in statistical threshold techniques used. ROI analyses often do not include a correction for multiple comparisons, as is standard practice for whole-brain, voxel-wise VBM studies. Thus, MTL effects may be present, but weaker, than some of the neocortical fitness-effects observed in whole-brain VBM studies.

Three cross-sectional studies have used diffusion-tensor imaging to examine the relationship between white matter integrity and fitness in older adults. Measuring the degree and directionality of the diffusion of water, diffusion tensor imaging provides insight to the microstuctural integrity of brain tissue (Basser and Pierpaoli, 1996; Le Bihan, 2003). The most common metric reported in diffusion studies is fractional anisotropy, which measures the primary direction of diffusion, considered to represent myelination, fiber density, and axonal diameter. In a study with young adults and older adults, a positive correlation was reported between estimates of cardiorespiratory fitness (based on non-aerobic measures such as age, BMI, gender, and results of an activity questionnaire) and fractional anisotropy values in the uncinate fasciculus and cingulum (Marks et al., 2007). A follow-up study in older adults indicated that VO_2_ peak was positively associated with fractional anisotropy in the middle and posterior segments of the cingulum bundle (Marks et al., 2011). In another study with older adults, VO_2_ peak (and total time on treadmill) correlated with fractional anisotropy and radial diffusivity measures in the body and genu of the corpus callosum (Johnson et al., 2012). Thus, although the current diffusion studies show limited regional consistency, most likely due to methodological differences (ROI vs. whole-brain), the results indicate that fitness has a positive impact on white matter integrity.

Brain structure has also been found to mediate the positive association between cardiorespiratory fitness and cognition in older adults. In one study, prefrontal cortical volume mediated the positive association between cardiorespiratory fitness and executive functions. Specifically, right inferior frontal gyrus volume mediated the relationship between a measure of cognitive interference on the Stroop task and VO_2_ max, whereas bilateral middle frontal gyrus volume mediated the relationship between accuracy on a spatial working memory task and VO_2_ max (Weinstein et al., 2012). In another study, volume of the caudate nucleus (an important node of frontal-subcortical networks) mediated the relationship between VO_2_ max and task switching accuracy (Verstynen et al., 2012). Thus, there is accumulating evidence that frontal-subcortical regions mediate the relationship between cardiorespiratory fitness and performance on tasks tapping executive functions, although this relationship has not been observed in all cases (e.g., the flanker task; Verstynen et al., 2012).

In a similar vein, hippocampal volume has been shown to mediate the relationship between aerobic fitness and spatial memory (Erickson et al., 2009; Szabo et al., 2011). In one of these studies, hippocampal volume was also associated with reductions in a self-report measure of frequency of forgetting everyday items, such as names, faces, and directions (Szabo et al., 2011). Together, these studies linking fitness, brain, and cognition are compelling, given that they suggest a positive link between cognitive functions and brain regions known to decline with age.

Data in AD are presently limited, but initial reports suggest a positive association between cardiorespiratory fitness and brain structure in patients with cognitive decline attributable to AD. For instance, VO_2_ peak correlated with global measures of white matter and whole brain volume as measured with VBM in AD patients (Burns et al., 2008). In a follow-up report, ROI analysis revealed that cardiorespiratory fitness was positively associated with hippocampal white matter and parahippocampal white and gray matter in patients with early stage AD (Figure 3B; Honea et al., 2009). In the only longitudinal study published to date, decline in VO_2_ peak over a 2-year period in AD patients was associated with volume reductions in parahippocampal gyrus and inferior temporal gyrus, as well as bilateral insula, right lingual gyrus and right putamen (Vidoni et al., 2012).

Recent advances in PET imaging have enabled *in vivo* detection of beta-amyloid plaques and neurofibrillary tangles, which are the hallmark signs of AD neuropathology. Using Pittsburgh Compound B (PiB), a tracer known to accumulate in regions encumbered with amyloid deposits, researchers have demonstrated that in cognitively normal older adults lower levels of physical activity (assessed using a self-report questionnaire) are associated with increased amyloid burden in cortical regions (the measure was collapsed across ROIs including prefrontal cortex, gyrus rectus, lateral temporal cortex, and precuneus (Liang et al., 2010). In a follow-up study with a larger sample, these results were replicated and further refined to show that lower levels of physical activity (assessed using a self-report questionnaire) were associated with elevated cortical PiB in APOE ε4 carriers (a genetic risk factor for AD), but not in non-carriers (Head et al., 2012).

### Intervention studies

The most powerful evidence supporting a relationship between cardiorespiratory fitness and brain health is derived from aerobic exercise intervention studies. To date, four studies have been published that have used an aerobic exercise intervention and pre- and post-intervention structural MRI (see Table 1). All studies used walking (with varying duration and intensity levels) as the primary type of exercise training in at least one study group, targeting enhanced cardiorespiratory fitness as the main training goal. Each of these studies recruited sedentary subjects (e.g., not more than two bouts of physical activity >30 min in previous 6 months; Voss et al., 2010b) on the assumption that improvements in cardiorespiratory fitness associated with aerobic exercise intervention are most likely to occur in these subjects, and therefore, these studies are most likely to demonstrate cardiorespiratory fitness-related brain changes.

The intervention studies in older adults indicate that aerobic exercise positively impacts brain structure (again, most frequently assessed using VBM). For example, increased volume of the anterior cingulate cortex, supplementary motor cortex, right inferior frontal gyrus, left superior temporal gyrus and anterior corpus callosum was reported in an aerobic training group (exercising at up to 70% of their heart rate reserve; 16.1% training increase in peak VO_2_) relative to a non-aerobic stretching control group (5.3% training increase in peak VO_2_; Colcombe et al., 2006). Importantly, the enhanced cardiorespiratory fitness and brain effects were observed after as few as 6 months of exercise (three 1-h sessions per week).

Another study similarly found that increases in physical activity (assessed with a questionnaire) resulting from exercise training over a 6-month period were associated with gray matter volume increases in the cingulate gyrus (including both anterior and posterior cingulate cortex), left superior frontal gyrus, left medial parietal cortex, and regions of occipital cortex (Ruscheweyh et al., 2011). These findings were not impacted by aerobic exercise intensity, and were similar for a walking (50–60% maximum exertion) and gymnastics (30–40% of maximum exertion) intervention. Ruscheweyh et al. (2011) also observed improved episodic memory (word list learning) with increased physical activity and observed a stronger association between increases in memory performance and physical activity in the low intensity exercise group relative to the medium intensity group. No physical activity- or performance-associated changes in the medial temporal lobes were observed. However, there was evidence that the association between increased physical activity and memory may be mediated by anterior cingulate cortex volume, as the relationship became non-significant when cingulate cortex volume was included in the regression model. The finding that lower intensity aerobic exercise elicited stronger cognitive effects than moderate intensity exercise was not expected, and certainly the exercise program was nowhere near the training intensity and duration that might elicit detrimental cardiorespiratory effects (O'keefe et al., 2012; Patil et al., 2012). However, it should be noted that neither training program elicited changes in their measure of cardiorespiratory fitness (measured with the lactate step test) in their participants, in contrast to other studies using the gold standard (peak VO_2_). The results are nonetheless noteworthy because the data suggest that increases in physical activity, even at low intensity, may positively impact brain structure and memory performance. However, the lack of training-related change in the measure of cardiorespiratory fitness suggests the results should be interpreted with caution.

Although the whole brain VBM studies discussed above did not report any significant findings in the MTL, studies using an ROI approach suggest that aerobic exercise positively impacts the structure of the MTL in older adults. After 1 year of training, older adults participating in aerobic training showed significant increases in VO_2_ max (7.8%) relative to the stretching control group (1.1%). Collapsing across all subjects, change in cardiorespiratory fitness (VO_2_ max) was positively associated with hippocampal volume. Furthermore, aerobically trained older adults demonstrated an approximately 2% increase in hippocampal volume (left and right), whereas a *decrease* of roughly 1.4% was observed in the stretching control group (Figure 3C; Erickson et al., 2011). These data are particularly striking given that the volumetric increases more than offset the annualized age-related MTL volume loss of 1–2% in this age group (Raz et al., 2004, 2005). The volumetric changes were specific to the anterior hippocampus, with no changes evident in posterior regions of the hippocampus. Furthermore, changes in serum levels of brain-derived neurotrophic factor, a growth factor involved in neurogenesis, were associated with increases in left and right anterior hippocampal volume in the aerobic training group, but not in the stretching control group. Performance on a spatial working memory task was positively associated with hippocampal volume, although improved spatial working memory was not directly linked to aerobic training. Taken together, the results of this study fit well with findings in the animal literature that show a relationship between physical activity, the MTL, and spatial memory performance, and provide some of the strongest evidence supporting a relationship between cardiorespiratory fitness, hippocampal volume, and memory performance in humans.

Only one exercise intervention study has examined white matter structure using diffusion tensor imaging (Voss et al., 2012). In this study, healthy older adults participated in a 12-month exercise-training program (three 40 min sessions/week) and were assigned to either an aerobic walking condition or a non-aerobic condition (stretching, toning, and balance exercises). Aerobic exercise increased a composite measure of cardiorespiratory fitness (average of VO_2_ max based on graded treadmill test and Rockport one-mile walk test) by 14.6%, whereas stretching, toning, and balance exercises increased cardiorespiratory fitness by 6.7%. There were no differences in white matter integrity as a function of training condition, although a trend for greater fractional anisotropy in a prefrontal ROI was observed for aerobically trained older adults relative to the non-aerobic group. Furthermore, there was a positive relationship between magnitude of change in cardiorespiratory fitness and fractional anisotropy values in prefrontal, parietal, and temporal ROIs in aerobically trained older adults. These effects were not observed in the non-aerobic training condition.

### Summary

The literature indicates that cardiorespiratory fitness is associated with brain volume and white matter integrity in healthy older adults. Regions showing the strongest relationship appear to be anterior cingulate cortex, lateral prefrontal cortex, and lateral parietal regions. Whereas whole brain studies have shown inconsistent effects in the MTL, ROI studies provide evidence that cardiorespiratory fitness is positively associated with structural integrity of the MTL in older adults. Although the data are currently limited, there is evidence from a cross-sectional study that physical activity thought to enhance cardiorespiratory fitness is associated with lower levels of AD-related neuropathology in individuals at risk for the disease. Finally, patients with mild AD show a positive association between brain structure and cardiorespiratory fitness. Results of the four exercise training studies implementing structural MRI were consistent with cross-sectional assessments, suggesting that cardiorespiratory fitness may contribute to healthy brain aging. Additional studies are necessary to clarify the exercise intensity required to observe changes in the aging brain and whether exercise interventions are effective in slowing neural decline in cognitively compromised populations.

## Linking cardiorespiratory fitness to brain function

Whereas T-1 weighted imaging and diffusion imaging offer insight into brain structure and structural connectivity, fMRI offers insight into neural activity and functional connectivity (Logothetis et al., 2001; Goense and Logothetis, 2008). Although fMRI has been available for over two decades, interest in the relationship between cardiorespiratory fitness and brain function in aging is more recent, with nine of the 10 identified functional brain imaging studies published within the last 2 years. Below we review published fMRI activation and connectivity studies (no PET studies were identified; Table 2).

### Cross-sectional studies

**Figure 4:**
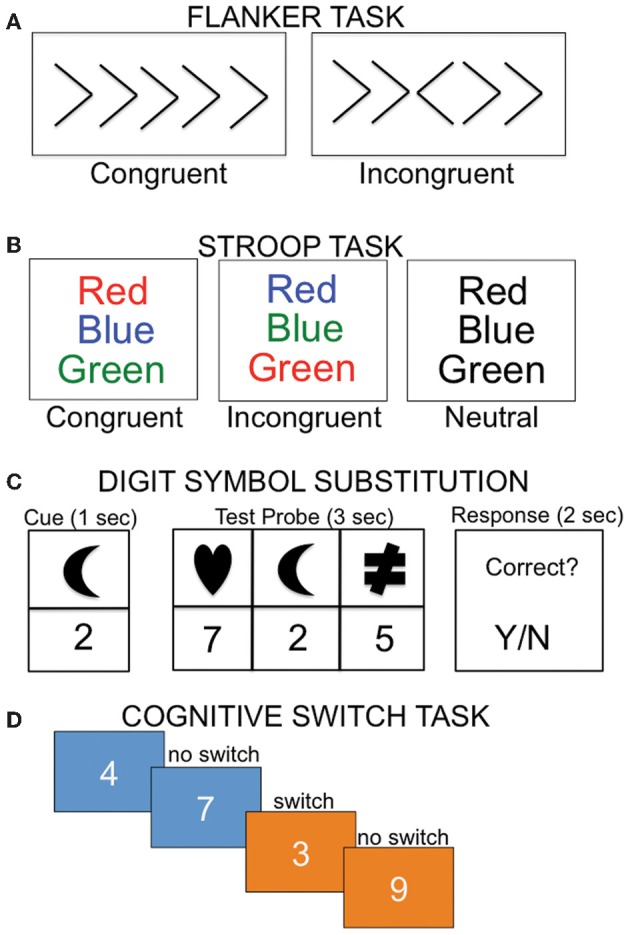
**Examples of cognitive tasks implemented in imaging studies examining the relationship between cardiorespiratory fitness and the brain. (A)** During the flanker task, subjects must indicate the direction of the center arrow, with the incongruent condition requiring additional cognitive control relative to the congruent condition. **(B)** In the Stroop task, participants are instructed to read the color of the print. In the incongruent condition, participants must inhibit the automatic response of reading the word. **(C)** In the digit symbol task, participants are required to map the associated number-shape pairs. **(D)** During the cognitive switching task, subjects are asked to judge whether a number is odd or even when the background is blue, or bigger or smaller than five when the background is orange.

**Figure 5:**
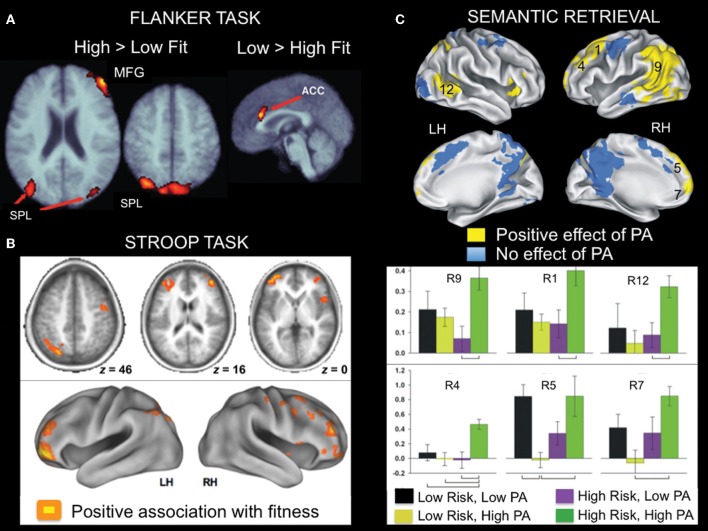
**Examples of fMRI studies showing a positive relationship between brain activity and aerobic fitness across a variety of cognitive tasks. (A)** Lateral fronto-parietal regions showed increased activation, whereas anterior cingulate cortex showed reduced activation during flanker task performance as a function of fitness in older adults. This pattern was evident in both a cross-sectional and an exercise intervention training study. From Colcombe et al. (2004), Figure 2, p. 3318. Adapted with permission. **(B)** During Stroop task performance, increased activation in lateral fronto-parietal regions was associated with aerobic fitness. From Prakash et al. (2011), Figure 4, p. 9. Adapted with permission. **(C)** Brain regions showing greater activation during famous name recognition as a function of physical activity level (PA) in older adults at increased risk for development of AD. The numbers associated with the activation cluster on the surface renderings correspond to the bar graphs presented below. From Smith et al. (2011a), Figures 3, 4, p. 640-641. Adapted with permission. ACC, anterior cingulate cortex; LH, left hemisphere; MFG, middle frontal gyrus; RH, right hemisphere; SPL, superior parietal lobe.

Colcombe and colleagues were the first to examine the association between cardiorespiratory fitness (estimated VO_2_ max using Rockport one mile walk test) and fMRI activity using a modified version of the Ericksen flanker task (Figure 4A; Colcombe et al., 2004). In a cross-sectional study, high cardiorespiratory fitness older adults showed increased activity in right middle frontal gyrus, bilateral superior parietal cortex, and visual cortex relative to low cardiorespiratory fitness older adults (Figure 5A), which was attributed to enhanced recruitment of fronto-parietal networks that bias attention to visual information and visual cortex activity. Low cardiorespiratory fitness older adults showed increased activity in anterior cingulate cortex (Figure 5A), a region sensitive to response conflict. Cardiorespiratory fitness was also linked to enhanced cognitive performance.

In another study that examined executive control using a modified Stroop task (Figure 4B), cardiorespiratory fitness (composite measure: average of VO_2_ max based on graded treadmill test and Rockport one-mile walk test) was associated with greater activation in bilateral middle and inferior frontal gyrus and left superior parietal cortex (Figure 5B; Prakash et al., 2011). Cardiorespiratory fitness was associated with faster response times in the incongruent condition, in which there was a mismatch between the word and the color in which the word is printed. Taken together, these studies show a consistent impact of cardiorespiratory fitness on activity in fronto-parietal regions that mediate enhanced cognitive control when there is processing conflict.

Another study (Rosano et al., 2010) reported an association between self-reported physical activities thought to enhance cardiorespiratory fitness (such as walking) and brain function in the context of a processing speed task. Participants were scanned 2 years after an exercise intervention (combined aerobic, strength, and flexibility training) or a successful aging intervention that did not include exercise (discussion of health-related topics, such as nutrition, medications, etc). During a digit symbol substitution task adapted for fMRI (Figure 4C), increased bilateral activity in superior/medial frontal gyrus was observed in the persistent physical activity group (exercise intervention and continued subsequent physical activity) relative to the persistent sedentary group (successful aging intervention and continued sedentary lifestyle). The physically active group performed the task more accurately and with faster response times.

A relationship between physical activity and brain function has also been observed during semantic memory retrieval. When older adults were asked to retrieve famous names, self-reported physical activities thought to be associated with cardiorespiratory fitness were linked with increased activation in several fronto-parietal regions including bilateral middle/superior frontal gyrus, bilateral medial frontal gyrus/supplementary motor area, left lateral parietal cortex/medial parietal cortex/middle temporal gyrus (Smith et al., 2011a). This effect was more pronounced in participants at risk for AD (Figure 5C), as defined by the presence of an ApoE ε 4 allele. In a follow-up study, increased left caudate nucleus activity was observed in MCI patients as a function of self-reported physical activity (Smith et al., 2011b). Interestingly, some of the same fronto-parietal regions showing a relationship with fitness during cognitive control tasks were also identified in the semantic retrieval task (Smith et al., 2011a). It is likely that the consistency in regions that show a relationship between fMRI activity and fitness reflects the shared cognitive component processes across these various tasks.

Two task-related fMRI studies have examined the relationship between cardiorespiratory fitness and motor-related activity. One study (Godde and Voelcker-Rehage, 2010) examined how a composite measure of cardiorespiratory fitness (including submaximal estimate of VO_2_ with a graded treadmill protocol, speed, agility, balance, coordination, flexibility, grip force) was related to brain activation while subjects imagined walking forward or backward. During imagined backward walking, high-fit older adults relative to low-fit older adults showed increased activation in supplementary motor area and ventral premotor cortex and reduced activation in several regions including right dorsolateral prefrontal cortex and left middle temporal gyrus. This pattern of activity was thought to reflect increased cognitive processing load in low-fit older adults, who may require more cognitive resources to activate internal motor representations than the high-fit older adults. In another study (McGregor et al., 2011), young adults and physically active older adults (assessed by questionnaire) suppressed activity in the ipsilateral motor cortex (as evident by deactivations) during right-handed motor movements, whereas sedentary older adults showed a failure to suppress activity (i.e., activations). Thus, fitness may be associated with reduced age-related decline in cross-hemisphere motor inhibition.

Finally, in the only functional connectivity study published to date, VO_2_ max was associated with connectivity in a subset of regions within the default-mode network (Voss et al., 2010a). The default mode network is engaged when individuals are left to think to themselves or during low-level cognitive tasks. The default mode network shows age-related alterations (Damoiseaux et al., 2008; Sperling et al., 2009) and is further disrupted in AD (Sperling et al., 2010), with amyloid deposition patterns demonstrating considerable overlap with the default mode network (for review, see Buckner et al., 2008). Voss et al. (2010a) found that cardiorespiratory fitness in elderly adults was positively correlated with functional connectivity between posterior cingulate cortex and anterior cingulate cortex, posterior cingulate cortex and left middle frontal gyrus, and parahippocampal gyrus and left middle frontal gyrus, among other regions. They also examined whether connectivity between regions of the default mode network mediated the relationship between cardiorespiratory fitness and performance on three tasks of executive function: task switching (Figure 4D), the Wisconsin Card Sorting Task, and spatial working memory. Posterior cingulate—anterior cingulate cortex connectivity mediated the relationship between cardiorespiratory fitness and perseverative errors on the Wisconsin Card Sorting Task, whereas middle temporal gyrus—middle frontal gyrus connectivity mediated the relationship between cardiorespiratory fitness and local switch cost (defined as difference in response time when the preceding trial consisted of the same task (no-switch) compared to a different task (switch). The results for the spatial working memory task were less clear. Connectivity between one pair of ROIs (middle frontal gyrus and anterior cingulate cortex) mediated the relationship between cardiorespiratory fitness and response time in the positive direction, whereas connectivity between another pair (parahippocampal gyrus and middle frontal gyrus) mediated the relationship between cardiorespiratory fitness and response time in the negative direction.

### Intervention studies

Three intervention studies have examined the impact of aerobic exercise training on brain function in older adults (see Table 2). Colcombe et al. (2004) were the first to demonstrate functional brain changes associated with exercise training. Training consisted of a 6-month exercise intervention, with the cardiorespiratory group training at up to 70% of heart rate reserve, with an observed 10.2% increase in VO_2_ max relative to a 2.9% increase in a stretching control group. Similar to the results of their cross-sectional study, they observed increased activity in lateral fronto-parietal regions and decreased activity in anterior cingulate cortex (region associated with response conflict monitoring) during flanker task performance in a group of aerobically trained older adults relative to a stretching control group (Figure 5A). Cardiorespiratory fitness was also linked to performance on the flanker task.

A more recent study (Voelcker-Rehage et al., 2011) compared the impact of two active training programs in older adults, aerobic training (walking) and coordination training, to a stretching/relaxation training control intervention, each of which met three times per week for 12 months. Another innovative feature of this study was that participants completed fMRI while performing a flanker task at baseline, 6 months, and 12 months. This multiple time-point design allowed researchers to assess the time course of exercise-associated changes in brain activation, such as whether exercise training effects plateau at 6 months or continue to increase at 12 months. Consistent with Colcombe et al. (2004), aerobic training resulted in decreased activation in anterior cingulate cortex during incongruent trials. Additionally, decreased activity was observed in left middle frontal gyrus, left parahippocampal gyrus, and left middle and superior temporal gyrus. Thus, in contrast to the pattern observed in cross-sectional fMRI studies and Colcombe et al. (2004), reductions in activation were predominant with aerobic training, while controls showed increased activation in these same regions following the stretching/relaxation intervention. On the other hand, coordination training resulted in increased activation during incongruent flanker trials in cortical regions such as inferior frontal gyrus and superior parietal regions, as well as subcortical regions including the caudate nucleus and thalamus. Both patterns (increased activation with coordination training; decreased activation with aerobic training) were evident at 6 months and continued in their respective direction at the 12-month assessment; that is, the effects did not plateau at 6 months. The authors suggested that the decreased prefrontal activation observed in aerobic training reflected “a reduced need for compensation or increased cognitive control” whereas coordination training reflected “more automated motor responses and more effective processing and integration of visual-spatial information” (pg. 10). This study highlights one of the primary challenges associated with functional brain imaging: determining whether increased or decreased activation represents an optimal pattern of neural functioning.

Finally, Voss et al. (2010b) examined aerobic exercise-associated changes in functional connectivity in the default network and two executive control networks: a frontal-insular network and a frontal-parietal network. They examined functional activation in older adults during passive viewing tasks at baseline, following 6 months, and following 12 months of aerobic exercise training (walking) or flexibility, toning, and balance training. They focused on regions that show age-related changes in connectivity relative to young adults. In comparison to non-aerobic training, 12 months of aerobic training led to increased connectivity in MTL, parietal, and frontal regions including enhanced connectivity between parahippocampal gyri and middle temporal gyrus, parahippocampal gyri and bilateral inferior parietal cortex, and left middle frontal gyrus and middle temporal gyri. Changes in the two executive control networks also showed some evidence favoring the aerobic training group, as nonsignificant trends for increased connectivity were observed within prefrontal regions (frontal-insular network) and between insula and lateral occipital cortex (frontal-parietal network). Interestingly, functional connectivity between parahippocampal gyrus and lateral occipital cortex and middle frontal gyrus and middle temporal gyrus, both pairs within the default mode network, was associated with performance on neuropsychological measures of executive functions. It is somewhat surprising that performance on executive tasks was most strongly related with connectivity within the default mode network (Voss et al., 2010a report a similar finding), which is typically linked to episodic or autobiographical memory (Daselaar et al., 2009), self-referential processing, and future thinking (Buckner et al., 2008), rather than with connectivity in frontal-parietal and frontal-insular networks, which are typically associated with cognitive control. It is possible that the significant association between the default mode network and executive functions in the older adults reflects age-related dedifferentiation, as Voss et al. note that the default mode network in older adults tended to show increased connectivity with nodes of both the frontal-insular network and frontal-parietal network. Regardless, the findings provide yet another example of aerobic exercise impacting brain function and associated performance on neuropsychological tasks measuring executive functions.

### Summary

Despite heterogeneity in the cognitive tasks implemented, there is consistency in the brain regions in older adults that show a positive relation between cardiorespiratory fitness and task-induced activation. These regions include the anterior cingulate cortex, lateral prefrontal regions, and lateral parietal regions, and associations with cardiorespiratory fitness were evident during canonical tasks of executive control (e.g., stroop, flanker task), a semantic memory retrieval task (which also requires executive control processes), and functional connectivity during passive viewing tasks. Importantly, these fronto-parietal regions appear to be the same regions showing structural associations with cardiorespiratory fitness. The majority of cardiorespiratory fitness-related patterns were increases in neural activity, with the exception of anterior cingulate cortex, which was associated with decreased activity in multiple studies. The decreased activity in this region was attributed to a reduction in neural resources necessary to resolve response conflict. The relationship between cardiorespiratory fitness and MTL function was less reliable for the fMRI studies, although this may be attributable to the lack of tasks implemented during fMRI that would be expected to elicit activation in MTL regions. There was also evidence to suggest a positive association of cardiorespiratory fitness with brain function in individuals with genetic risk for AD and those with mild cognitive impairment, but further evidence is needed as these studies did not directly measure cardiorespiratory fitness, but rather inferred fitness from self-reported measures of physical activity. We did not identify any fMRI studies examining the relationship between cardiorespiratory fitness and brain function in AD patients. This may in part be due to the challenges of conducting task-based fMRI in individuals who may not be able to learn and remember task instructions and may become confused during the course of the study. Given the cognitive impairment associated with AD and the cognitive demands for task performance during fMRI, one would likely need to test patients in the early stages of the disease, using simple cognitive tasks, or relying exclusively on resting-state fMRI (which does not require task performance).

## Discussion

The extant data from both structural and functional neuroimaging studies support the notion that cardiorespiratory fitness links to neural integrity in older adults. Despite the differences in imaging modality and cardiorespiratory fitness assessment methods, there is consistency in the brain regions showing a relationship with cardiorespiratory fitness, as both cross-sectional and aerobic exercise training studies using functional and structural imaging have demonstrated a relationship with cardiorespiratory fitness in regions such as anterior cingulate cortex, medial and lateral prefrontal cortex, and lateral parietal regions. The relationship between cardiorespiratory fitness and structural integrity of the MTL is inconsistent in whole-brain VBM studies, although several ROI studies support the tenant that cardiorespiratory fitness is correlated with the structural integrity of the MTL. It is possible that the inconsistent MTIL results are attributable to the differences in statistical thresholding procedures implemented (e.g., corrections for multiple comparisons) for whole-brain relative to ROI studies. Cardiorespiratory fitness-MTL effects have not consistently been found in fMRI studies, likely due to the lack of studies using tasks that reliably elicit MTL activation.

A remaining question is whether the consistent association of cardiorespiratory fitness with fronto-parietal regions observed across various tasks is attributable to shared cognitive component processes across these tasks. For instance, executive control processes may be an important component driving performance in both the flanker task and tasks of semantic memory retrieval. Alternatively, is there something about the neurobiological organization of frontal and parietal cortex (or its tendency to decline with aging) that makes these regions more sensitive to changes in cardiorespiratory fitness? Disentangling this issue will require the use of tasks tapping different cognitive functions during fMRI in the same group of participants. For instance, implementing a flanker task (executive control), face-name memory task (episodic memory), and face/scene viewing task (visual perception) could potentially identify brain regions that are sensitive to cardiorespiratory fitness across tasks and regions that may show domain-specific relationships with cardiorespiratory fitness. We are not aware of any fitness studies that have implemented such an approach. Unfortunately, a quantitative meta-analytic approach to identify the exact neural coordinates of cardiorespiratory fitness was not possible in the present review, as the majority of studies did not present a table of significant cardiorespiratory fitness-brain region relationships in standard neuroanatomical space, as is required for such analysis (Turkeltaub et al., 2002). As the literature continues to grow, it will be critical to assess the exact spatial overlap of the neural correlates of cardiorespiratory fitness. We recommend that future studies provide this information to allow for quantitative meta-analytic approaches to neuroimaging data (Hayes et al., 2012), which would facilitate identification of general or task-specific neural correlates of cardiorespiratory fitness.

The evidence in humans that cardiorespiratory fitness and aerobic exercise impact the brain is consistent with longstanding reports in the animal literature (for review, see Cotman and Berchtold, 2002; Dishman et al., 2006; Cotman et al., 2007; Van Praag, 2009). However, little is known about the mechanisms that underlie these effects in humans. Studies in animal models have addressed neurobiological mechanisms by which physical activity (usually a rodent running on a wheel) impacts the brain, including neurogenesis, angiogenesis, and mRNA transcription, but given the different scale at which neural integrity is assessed in humans, it would be difficult to assume similar mechanisms are at work in humans (cf. Thomas and Baker, 2013). Therefore, it will be important for future studies to more closely integrate results from animal models and humans to elucidate the specific neurobiological mechanisms of cardiorespiratory fitness related neuroplasticity. An example of such an approach is provided by Pereira et al. (2007), who implemented similar methods in both mice and humans (young adults). They examined the effects of 12 weeks of aerobic exercise (four times per week for 40 min) using cerebral blood volume (CBV) maps derived from pre- and post-gadolinium contrast enhanced T1-weighted images. In mice, exercise was associated with greater CBV in the dentate gyrus, which was subsequently linked to post-mortem indicators of neurogenesis in that region. In young adults, changes in VO_2_ max were associated not only with CBV changes in the dentate gyrus, but also with performance on a verbal learning task. Although the study has yet to be replicated [a related study reported increased hippocampal blood flow and connectivity in six aerobically training older adults relative to five control participants, although post-training measures of cardiorespiratory fitness or physical activity were not reported (Burdette et al., 2010)], it provides support for the notion that CBV may be an imaging correlate of neurogenesis in humans, and provides evidence for the notion that cardiorespiratory fitness may enhance neurogenesis in the MTL and, as a result, boosts episodic memory performance.

It is noteworthy that animal studies highlight the MTL (and in particular, the dentate gyrus) as the region most susceptible to effects of physical activity related to cardiorespiratory fitness, whereas in humans, the most consistent findings appear to localize to fronto-parietal regions. These neural findings mirror the cognitive findings, in which the impact of physical activity (wheel running) on behavior in rodents is most commonly associated with animal analogues of episodic memory tasks—that is, hippocampally-mediated tasks such as the Morris water maze or radial arm maze (Van Praag et al., 2005; Kronenberg et al., 2006; Nichol et al., 2009). In contrast, the strongest effects of cardiorespiratory fitness on cognition in humans are seen in tasks that are dependent on fronto-parietal regions and putatively rely on higher-order executive functions (Colcombe and Kramer, 2003). This apparent cross-species discrepancy in neural correlates of cardiorespiratory fitness may be attributable to the fact that few human studies have used tasks that are optimally sensitive to MTL function. The few human studies that have used spatial memory tasks, known to be hippocampally-dependent, have shown a positive relationship with cardiorespiratory fitness (Erickson et al., 2011; Holzschneider et al., 2012). Therefore, discrepancies in the results of animals and human studies may be due to differences in the neural substrates required for task performance. Future human studies examining the effect of cardiorespiratory fitness on a variety of episodic memory tasks sensitive to MTL function will thus be of particular interest.

The notion that exercise may mitigate age-related cognitive and neural decline is appealing for a variety of reasons, including that it is a readily available, low cost activity that could potentially contribute to improving or maintaining quality of life. We caution that exercise is not a cure for age or AD-related neural and cognitive decline, but that it may mitigate detrimental effects to some extent. It is important to note that the majority of studies conducted to date and reviewed here were cross-sectional, and many of the extant studies have been performed by the same group of researchers (Kramer, Erickson and colleagues). Additional intervention studies are needed to examine dose effects that yield the strongest impact on cognition and the brain, including comparisons of frequency, intensity, and duration of exercise training. Although we have focused exclusively on cardiorespiratory fitness, two recent reports have examined the impact of resistance training on cognition on the brain, and suggest that resistance training may positively impact cognitive performance and brain function (Liu-Ambrose et al., 2012; Nagamatsu et al., 2012). The results of Liu-Ambrose et al. (2012) are noteworthy because they suggest that different types of exercise training may impact different cognitive functions and distinct brain regions (Voelcker-Rehage and Niemann, 2013), although this was not directly examined in their study. Future large-scale studies that directly contrast different exercise programs may begin to clarify exercise-specific cognitive and brain changes. Furthermore, integration of multiple imaging modalities, including those outlined in this review as well as magnetic resonance spectroscopy, which images specific metabolites such as *N*-acetyl-aspartate that correlate with cardiorespiratory fitness (Erickson et al., 2012; Gonzales et al., 2013), may clarify the neurophysiological mechanisms supporting cardiorespiratory fitness-related cognitive changes. Interactive effects of physical activity with diet (Scarmeas et al., 2009) and genes (Leckie et al., 2012) further highlight the number of issues that remain to be addressed.

In conclusion, the extant data, which includes seven intervention studies, suggest that large-scale, low-cost behavioral interventions, such as exercise, have the potential to elicit enhanced cognitive performance and neuroplasticity in the aging brain. Data from AD are currently limited to two cross-sectional studies that show higher cardiorespiratory fitness is associated with larger brain volume in patients with mild AD. These findings suggest that cardiorespiratory fitness-related neuroplasticity may not be limited to cognitively intact populations, although definitive studies (exercise training with imaging outcome measures) have yet to be completed and intervention efficacy may be complicated by the presence of cardiovascular risk factors (Eggermont et al., 2006). Finally, there are data to suggest that these alterations in neural integrity can be achieved with low-intensity exercise programs (e.g., walking) and within a relatively short period of time (6 months). Both of these features could facilitate implementation and adherence to exercise programs for older adults, MCI and AD patients.

## Limitations

For our current purposes, we considered any neuroimaging study that included objective measures of cardiorespiratory fitness (maximal or sub-maximal testing, or equation-derived) or subjective self-report measures of physical activity (questionnaires), which as noted previously, rely on the inference that higher levels of activity are associated with higher levels of fitness. We note that cardiorespiratory fitness and physical activity may not have an exact one-to-one relationship, as cardiorespiratory fitness is likely impacted by multiple factors (e.g., genetics, nutrition), and therefore one could have high cardiorespiratory fitness but also be sedentary (low physical activity). However, data reveal a correlation between these distinct constructs across individuals (Bowles et al., 2004; McAuley et al., 2011), and both appear equally important in determining health-related outcomes (Blair et al., 2001). An implicit assumption in the intervention studies is that the primary brain benefits are derived from increased cardiorespiratory fitness; however, enhancement in other fitness domains, such as muscular endurance, also occurs. This is best illustrated by the fact that a non-aerobic flexibility training group, a common control group for aerobic training, nevertheless shows some evidence of increases in VO_2_ max (in some studies roughly 5%), albeit smaller than that typically seen for aerobically trained older adults (15–18% increase, e.g., Colcombe et al., 2006). Thus, any given type of exercise training (e.g., aerobic) can impact multiple types of fitness. These well-controlled studies may in fact be underestimating the impact of aerobic training on the brain because the control group is also showing training-related improvements in cardiorespiratory fitness. Additional factors such as genetics, nutrition, social engagement, and socio-economic and educational status are likely to influence brain aging as well, but are outside the scope of this review. A quantitative meta-analysis of the imaging data (e.g., Hayes et al., 2012) was precluded by the dearth of studies reporting whole-brain coordinates. This will be critical to examine the exact overlap of brain regions associated with cardiorespiratory fitness Finally, as noted at the outset of the current review, many of the studies to date have been cross-sectional, which limits the causal attribution of cardiorespiratory fitness to positively impact brain aging. Nevertheless, the available intervention studies support the results of the cross-sectional studies.

### Conflict of interest statement

The authors declare that the research was conducted in the absence of any commercial or financial relationships that could be construed as a potential conflict of interest.
